# Periodontitis induced by bacterial infection exacerbates features of Alzheimer’s disease in transgenic mice

**DOI:** 10.1038/s41514-017-0015-x

**Published:** 2017-11-06

**Authors:** Naoyuki Ishida, Yuichi Ishihara, Kazuto Ishida, Hiroyuki Tada, Yoshiko Funaki-Kato, Makoto Hagiwara, Taslima Ferdous, Mohammad Abdullah, Akio Mitani, Makoto Michikawa, Kenji Matsushita

**Affiliations:** 1 Department of Operative Dentistry, Endodontology and periodontology, School of Dentistry, Matsumoto Dental University, 1780 Hirookaobara, Shiojiri, Nagano 399-0781 Japan; 2 Department of Periodontology, School of Dentistry, Aichi Gakuin University, 2-11 Suemori-dori, Chikusa-ku, Nagoya 464-8651 Japan; 3 Department of Oral Disease Research, National Center for Geriatrics and Gerontology, 7-430 Morioka-cho, Obu, 474-8511 Japan; 4 Department of Physical Therapy, Graduate School of Medicine, Nagoya University, 1-1-20 Daikouminami, Higashi-ku, Nagoya 464-8601 Japan; 5 Department of Biochemistry, Graduate School of Medical Sciences, Nagoya City University, 1 Kawasumi Mizuho-cho, Mizuho-ku, Nagoya 467-8601 Japan

## Abstract

Periodontitis is a localized infectious disease caused by periodontopathic bacteria, such as *Porphyromonas gingivalis*. Recently, it has been suggested that bacterial infections may contribute to the onset and the progression of Alzheimer’s disease (AD). However, we do not have any evidence about a causative relationship between periodontitis and AD. In this study, we investigated by using a transgenic mouse model of AD whether periodontitis evoked by *P. gingivalis* modulates the pathological features of AD. Cognitive function was significantly impaired in periodontitis-induced APP-Tg mice, compared to that in control APP-Tg mice. Levels of Amiloid β (Aβ) deposition, Aβ40, and Aβ42 in both the hippocampus and cortex were higher in inoculated APP-Tg mice than in control APP-Tg mice. Furthermore, levels of IL-1β and TNF-α in the brain were higher in inoculated mice than in control mice. The levels of LPS were increased in the serum and brain of *P. gingivalis*-inoculated mice. *P. gingivalis* LPS-induced production of Aβ40 and Aβ42 in neural cell cultures and strongly enhanced TNF-α and IL-1β production in a culture of microglial cells primed with Aβ. Periodontitis evoked by *P. gingivalis* may exacerbate brain Aβ deposition, leading to enhanced cognitive impairments, by a mechanism that involves triggering brain inflammation.

## Periodontal disease is a risk factor for Alzheimer’s disease

Alzheimer’s disease is a scourge of longevity that will drain enormous resources from health budgets in the future. Unfortunately, the pathogenesis of the disease remains an enigma and there is no adequate treatment or prophylaxis for the disease. One key priority is to examine the modifiable risk factors that influence the development of dementia. A risk factor relationship between periodontal pathogens/periodontal disease and AD exists. In Alzheimer’s model mice infected with *Porphromonas gingivalis*, a type of periodontopathic bacterium, cognitive function was reduced and deposition of amyloid β peptide was increased. Moreover, inflammatory cytokines and bacterial endotoxin were increased in the serum and brain. Neuroinflammation may be caused by an increase in these inflammatory mediators, and the pathology of Alzheimer’s disease may be exacerbated. Since periodontal infections are treatable, treatment of periodontal diseases during this period may be effective for delaying the onset or progression of AD.

## Introduction

Periodontitis is a chronic inflammatory disorder in the oral cavity that causes destruction of supportive tissues including the alveolar bone around teeth, finally leading to tooth loss. A resident biofilm, so-called dental plaque, that forms in subgingival pockets evokes chronic inflammation in periodontal tissues and causes alveolar bone loss.^1^ Periodontitis develops due to the interaction between periodontal pathogenic bacteria and the host. One of the important periodontal pathogens, *Porphyromonas gingivalis*, is strongly involved in the onset and progression of periodontitis. *P. gingivalis* and its toxins, such as lipopolysaccharide (LPS) induce the production of proinflammatory cytokines and chemokines and promote inflammation in periodontal tissues. Studies have shown possible links between periodontitis and diseases including diabetes,^2,3^ atherosclerosis,^4,5^ osteoporosis,^6^ coronary heart disease,^7,8^ and Alzheimer’s disease (AD).^9–13^ Results of studies have shown intravascular infiltration of periodontitis-related bacteria and their spread to target organs, but the mechanisms by which the bacteria cause diseases are not fully understood.

AD is a brain disorder that affects a region of the brain that controls cognitive function and memory. In the initial stage of this disease, recent memory is affected, but long-term memory, learning, decision and communication capabilities are also gradually affected as the disease progresses.^14,15^ Lines of evidence have suggested that AD is caused by accumulation of amyloid β peptide (Aβ), leading to tauopathy accompanied by synaptic dysfunction and intracerebral inflammation, which is assumed to be caused by Aβ deposits.^16–18^ Although it has been reported that long-term administration of nonsteroidal anti-inflammatory drugs can prevent the occurrence of neurodegenerative diseases,^19^ it has been claimed in some reports that there is no such effect.^20^ In the immune response in the central nervous system, microglia play a central role.^21^ Microglia function in the elimination of waste products that have accumulated in the brain. These cells also produce cytokines, such as interleukin (IL)-1β, IL-6, and tumor necrosis factor-α (TNF-α), and reactive oxygen species, and they induce neurodegeneration in AD, indicating the possibility that they promote.^21,22^

It is possible that chronic inflammation that has developed in peripheral organs exacerbates the pathology of AD, and one of such chronic inflammatory diseases is periodontitis. Periodontal pathogenic bacteria spread throughout the body via blood vessels and airways. In addition, it is believed that inflammatory mediators, such as cytokines produced in periodontal tissues are carried to target organs in a hematogenous manner, exacerbating the inflammatory response in the organs.^23^ nterestingly, *P. gingivalis* is frequently detected in autopsied brain tissue of AD patients.^24^ These findings suggest that periodontal pathogenic bacteria may exacerbate the pathology of AD. In addition, several studies have shown correlations of periodontal diseases with cognitive function and AD.^9–13^ However, the molecular mechanism by which periodontitis is involved in AD pathogenesis has not been clarified.

In this study, we induced experimental periodontitis by *P. gingivalis* infection in the oral cavity of amyloid precursor protein (APP) transgenic mice and examined the effect of periodontitis on pathophysiology of AD in those mice.

## Results

### Development of periodontitis by *P. gingivali*s infection in APP-Tg mice

**Fig. 1:**
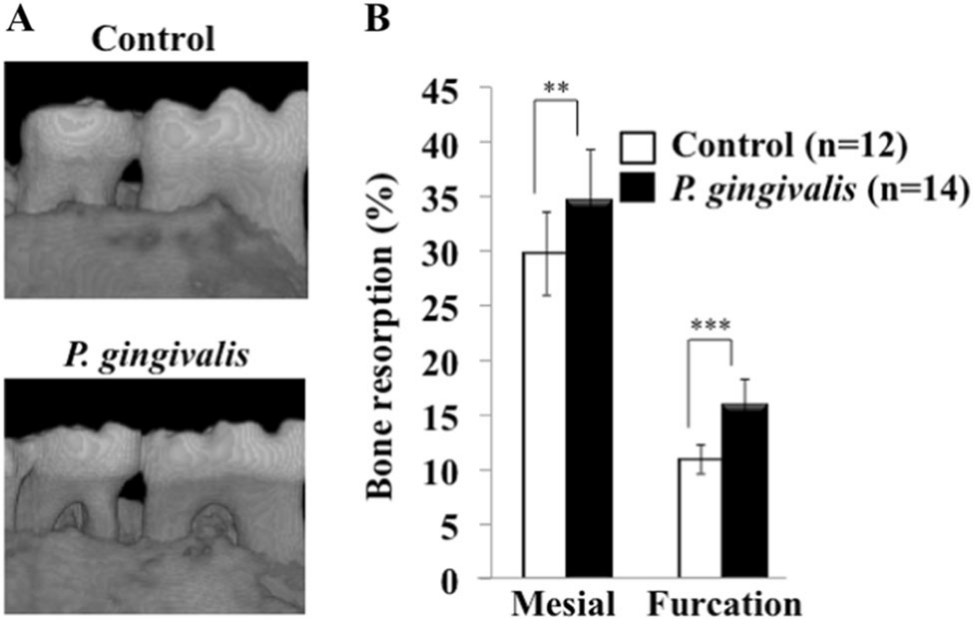
Evaluation of alveolar bone loss in *P. gingivalis*-infected APP-Tg mice. **a** Alveolar bone resorption (imaged by µ-CT). Control: Mice inoculated with CMC alone, *P. gingivalis*: Mice inoculated with CMC and *P. gingivalis*. **b** Alveolar bone resorption was evaluated in the furcation and mesial side of the second molar mandible. Results are expressed as means ± S.D., **p < 0.01, ***p < 0.001

**Fig. 2:**
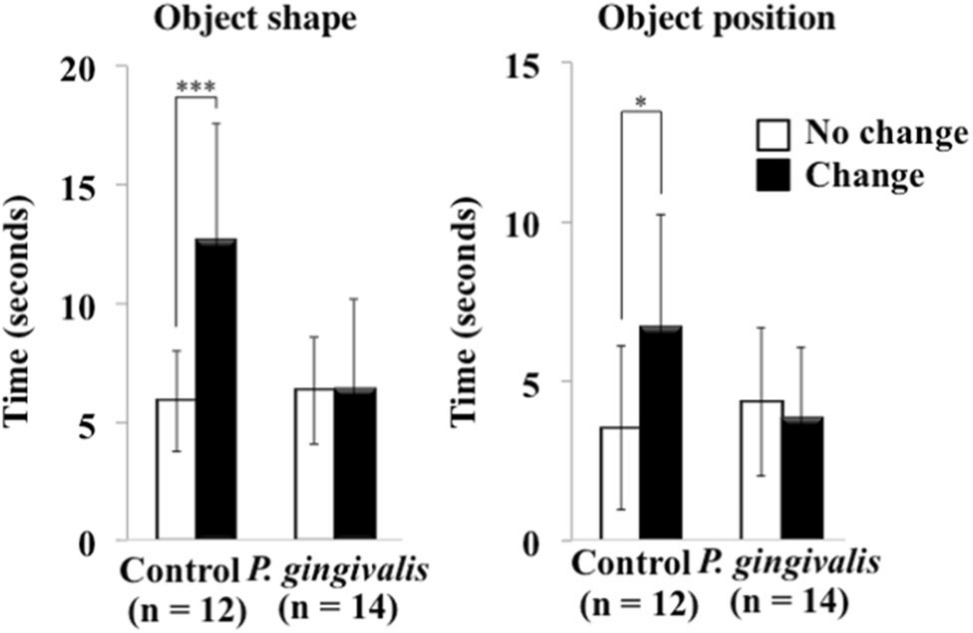
Evaluation of cognitive function in *P. gingivalis*-infected APP-Tg mice. Results are expressed as means ± S.D., *p < 0.05, ***p < 0.001

To develop experimental periodontitis in APP-Tg mice, we inoculated the mice with *P. ginigivalis* ATCC 33,277 mixed with carboxymethyl cellulose, which was delivered orally. After 5 weeks, we evaluated alveolar bone loss in the mice. As shown in Fig. 1, *P. gingivalis-*infected mice displayed significantly increased alveolar bone loss compared with that in control mice, as indicated by a decrease in relative amounts of bone around mandibular molars of infected mice. Alveolar bone resorption in the mesial side and furcation area of the μm^2^, the level of Aβ deposition was increased in the hippocampus of *P. gingivalis*-infected mice (19,182.2 ± 7334.9 μm^2^) (*P* = 0.0028, *F* = 0.82). The Aβ deposition area in the cortex in the *P. gingivalis*-infected group (4263.8 ± 3257.2 μm^2^) was larger than that in the control group (2658.2 ± 1312.6 μm^2^) but these values were not significantly different (*P* = 0.23, *F* = 0.041) (Fig. 2).

**Fig. 3:**
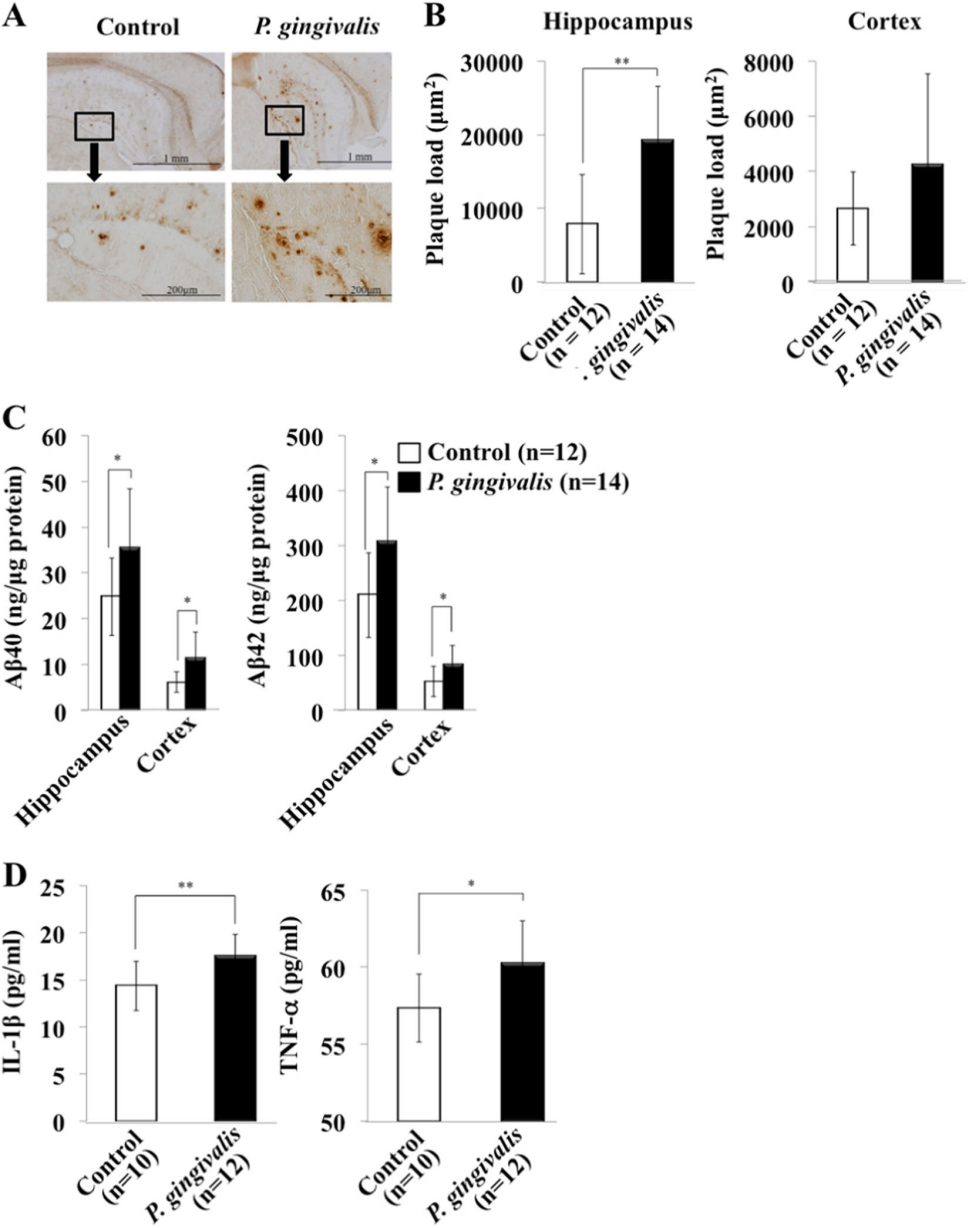
Amyloid plaque deposition in the cortex and hippocampus of APP-Tg mice. **a** Immunostaining of Aβ in brain sections. **b** Aβ loads in brain sections. Results are expressed as means ± S.D., **p < 0.01. **c** Aβ40 and Aβ42 in the cortex and hippocampus of *P. gingivalis-*infected APP-Tg mice. Concentrations of Aβ40 and Aβ42 in the extracts of brain were measured by ELISA. Control: Mice inoculated with CMC alone, *P. gingivalis*: Mice inoculated with CMC and *P. gingivalis*. Results are expressed as means ± S.D., *p < 0.05. **d** TNF-α and IL-1β in the brain in *P. gingivalis-*infected APP-Tg mice. Amounts of TNF-α and IL-1β in brain extracts were measured by ELISA. Results are expressed as means ± S.D., *p < 0.05, **p < 0.01

We next examined the concentrations of Aβ in the brains from *P. gingivalis*-infected APP-Tg mice. As shown in Fig. 3c, the brain levels of Aβ40 and Aβ42 in *P. gingivalis*-inoculated mice than in control mice. Production levels of Aβ40 and Aβ42 in the hippocampus in the control group were 24.8 ± 8.5, and 209.8 ± 77.1 ng/μg protein, respectively. On the other hand, those levels in the hippocampus in the *P. gingivalis-*infected group were 35.4 ± 13.1 and 307.1 ± 99.7 ng/μg protein, respectively. The values were significantly different (Aβ40: *P* = 0.047, *F* = 0.21; Aβ42: *P* = 0.025, *F* = 0.45) between the groups. Aβ40 and 42 production levels in the cortex in the control group were 6.16 ± 2.3 and 52.5 ± 27.6 ng/μg protein, respectively, and the levels in the cortex in the *P. gingivalis*-infected group were 11.4 ± 5.7 and 84.3 ± 33.1 ng/μg protein, respectively. The values were also significantly different (Aβ40: *P* = 0.011, *F* = 0.01; Aβ42: *P* = 0.031, *F* = 0.62) between the groups.

AD is caused by accumulation of Aβ due to intracerebral inflammation, and increased levels of proinflammatory cytokines, such as TNF-α and IL-1β were shown in AD patients. Therefore, we examined whether *P. gingivalis* infection affects the levels of TNF-α and IL-1β in the brains of APP-Tg mice. Quantification using ELISA showed that the brain levels of IL-1β and TNF-α were higher in the *P. gingivalis*-inoculated mice than in the control mice (IL-1β: 17.5 ± 2.3 μg/ml vs. 14.4 ± 2.6 μg/ml, *P* = 0.0062, *F* = 0.66; TNF-α: 60.2 ± 2.8 μg/ml vs. 57.3 ± 2.2 μg/ml, *P* = 0.016, *F* = 0.51) (Fig. 3d).

### Endotoxin concentrations in the brain and serum of *P. gingivalis-*infected APP-Tg mice

**Fig. 4:**
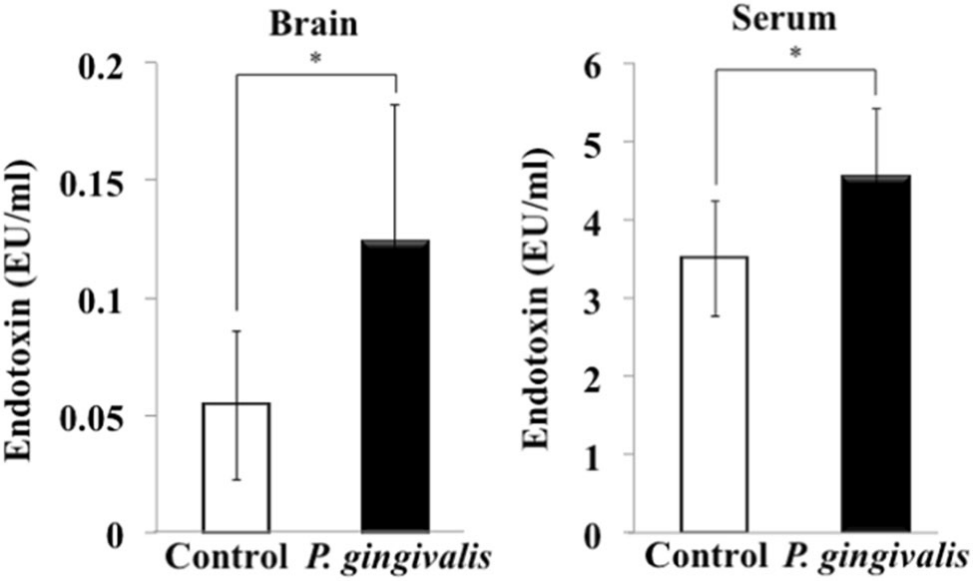
Concentrations of endotoxin in the brain and serum from *P. gingivalis*-infected mice. Levels of endotoxin in the brain and serum were measured by Limulus assay. Results are expressed as means ± S.D., *p < 0.05, **p < 0.01

We then examined the mechanism by which *P. gingivalis* infection enhanced brain inflammation. A recent study showed that *P. gingivalis* and its LPS were found at a high frequency in autopsied brain tissues of patients who died of AD; however, they were not found in normal human brain tissues. Therefore, we measured the concentrations of bacterial LPS in the sera and brains of *P. gingivalis*-inoculated APP-Tg mice. As shown in Fig. 4, although endotoxin was detected even in the sera and brains of control mice (3.51 ± 0.74 EU/ml and 0.05 ± 0.03 EU/ml, respectively), the levels were increased in the sera and brains of *P. gingivalis*-inoculated mice (4.54 ± 0.88 EU/ml and 0.12 ± 0.06 EU/ml, respectively) and the values were different (Sera: *P* = 0.033, *F* = 0.74; Brains: *P* = 0.022, *F* = 0.2).

### Induction of Aβ by stimulation with *P. gingivalis* LPS in neuronal cell cultures

**Fig. 5:**
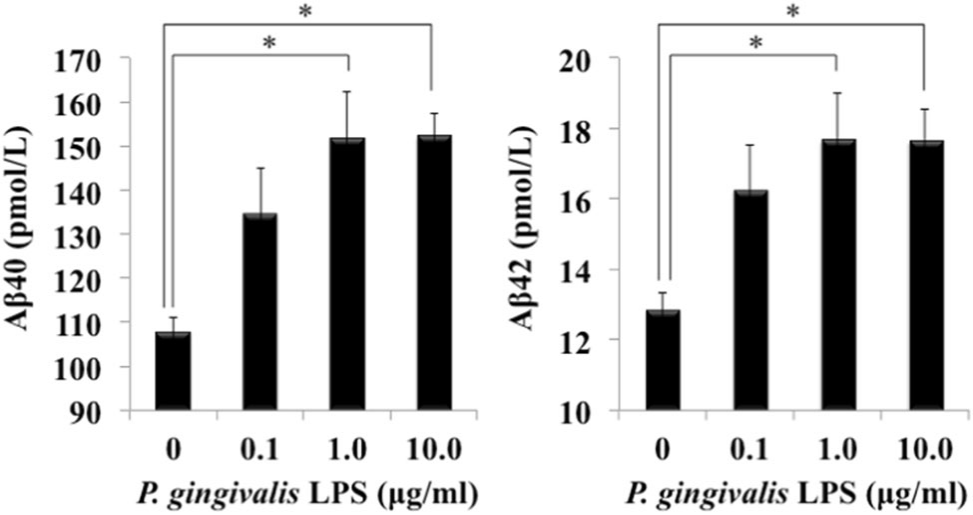
*P. gingivalis* LPS induces Aβ production in mouse neural cell cultures. Neural cells isolated from mouse brain were stimulated with *P. gingivalis* LPS for 24 h. Amounts of Aβ40 and Aβ42 in the media were measured by ELISA (*n* = 3, mean ± S.D., *p < 0.05)

To try to examine the mechanism by which *P. gingivalis* LPS enhances brain inflammation, we examined whether *P. gingivalis* LPS induces Aβ production in murine neuronal cell cultures. As shown in Fig. 5, Aβ40 and Aβ42 production levels were increased in the media in neuronal cell cultures stimulated with 1.0 (105.9 ± 3.4 pmol/L vs. 151.9 ± 3.4 pmol/L; *P* = 0.002, *F* = 0.084) and 10.0 µg/ml (105.9 ± 3.4 pmol/L vs. 152.3 ± 5.0 pmol/L; *P* = 0.00052, *F* = 0.65) of *P. gingivalis* LPS.

### Effect of *P. gingivalis* LPS on Aβ-induced cytokine production in microglia cell cultures

**Fig. 6:**
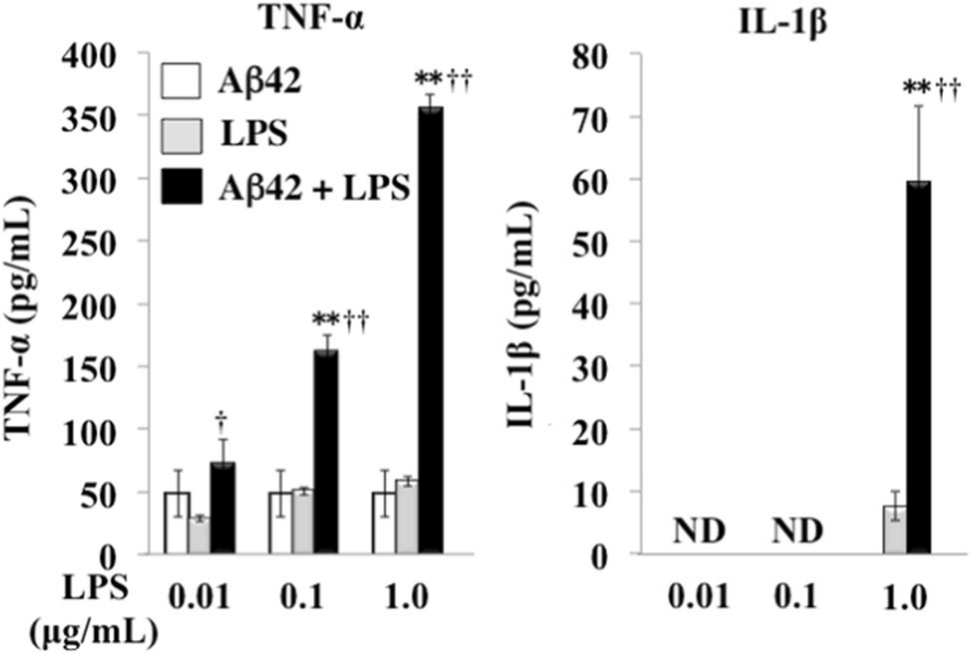
*P. gingivalis* LPS enhances Aβ-induced cytokine production in microglia cell cultures. Microglial cells were stimulated with 10 µM Aβ42 for 6 h and further stimulated with *P. gingivalis* LPS (0.01, 0.1, 1.0 μg/ml) for 24 h. Amounts of TNF-α and IL-1β in the media were quantified by ELISA (*n* = 3, mean ± S.D.). **p < 0.01 vs. Aβ 42 alone; ^†^ *p* < 0.05, ^††^ *p* < 0.01, vs. LPS alone

Aβ oligomers induce overproduction of proinflammatory cytokines, such as TNF-α and IL-1β. To determine the effects of *P. gingivalis* LPS on Aβ42-induced inflammatory responses in microglia, various concentrations of *P. gingivalis* LPS were added to microglial cell cultures primed with fibrillized Aβ42, and protein levels of TNF-α and IL-1β in the media were measured by ELISA. Small amounts of TNF-α were induced in the media from microglial cells primed by 10 µM Aβ42 and by stimulation with 0.01–1.0 µg/ml *P. gingivalis* LPS. However, by stimulation with 0.1–1.0 µg/ml *P. gingivalis* LPS, the production of TNF-α was significantly increased in the media from Aβ 42-primed microglia (Aβ42: 48.4 ± 18.5 pg/ml vs. Aβ42 + 0.1 µg/ml LPS: 162.6 ± 12.0 pg/ml, *P* = 0.0038, *F* = 0.51; Aβ42: 48.4 ± 18.5 pg/ml vs. Aβ42 + 1.0 µg/ml LPS: 355.7 ± 10.9 pg/ml, *P* = 0.0012, *F* = 0.54) (Fig. 6). On the other hand, IL-1β production was not induced in the media in Aβ42-primed microglial cell cultures. However, 1.0 μg/ml of LPS stimulation significantly enhanced IL-1β production in Aβ42-primed microglial cell cultures (LPS: 7.5 ± 2.3 pg/ml vs. LPS + Aβ42: 59.5 ± 12.3 pg/ml, *P* = 0.037, *F* = 0.28). These results suggest that *P. gingivalis* LPS may exacerbate brain inflammation in AD by activating microglia cooperatively with Aβ.

## Discussion

Recent epidemiological evidence has shown that periodontitis and its causative bacteria may also be associated with AD. However, these causative relationships have not been shown. In the present study, we demonstrated for the first time that periodontitis, caused by *P. gingivalis* infection, exacerbates the pathological features of AD in a murine model.

How does *P. gingivalis* infection exacerbate the pathological features of AD? Chronic inflammation in the brain is thought to play an important role in the etiology of AD and it is featured by the production of proinflammatory cytokines, such as TNF-α, IL-1β, and IL-6, by activated microglia.^19,25^ It was shown that Aβ fibrils can induce excess production of cytokines and neurotoxic mediators to activate microglia and can injure neurons.^26,27^ On the other hand, previous studies demonstrated that chronic bacterial infections, such as rheumatoid arthritis, leprosy, tuberculosis, syphilis, and osteomyelitis are often associated with the deposition of amyloid.^28^ The bacterial endotoxin LPS is a major inducer of inflammation and LPS may induce chronic neuroinflammation, Aβ accumulation/deposition, and impairment of cognitive function.^29^ A recent study showed that *P. gingivalis* LPS was found at a high frequency in autopsied brain tissues of patients who died of AD; however, it was not found in normal human brain tissues.^24^ It has also shown that *P. gingivalis* could enter the brain of ApoE knockout mice.^30^ For the mechanism of *P. gingivalis* entry into the brain, it has been shown that *P. gingivalis* has the ability to bind to E-selectin via its OmpA-like protein,^31^ and can invade cells by activation of intercellular adhesion molecule (ICAM)-1 and the small G-protein Rab5 in host cells.^32,33^ These lines of evidence suggest that *P. gingivalis* and its toxin probably spread by a hematogenous route and are transmitted into the brain via the blood–brain barrier (BBB). Besides the experiments using J20 mice, we also performed experiments to examine memory function, levels of serum cytokines and endotoxin, and endogenous brain Aβ levels in *P. gingivalis*-infected WT mice. The results of the novel objection test showed that cognitive function was impaired in mice of the *P. gingivalis* administration group compared with that in the control mice (Supplementary Fig. 1), but the brain Aβ level and serum TNF-α, and endotoxin levels were not different from those in the control group (Supplementary Figs. 2 and 3). A small amount of TNF-α was also found in the brain of WT mice, but there was no significant difference between the *P. gingivalis* administration group and control group (data not shown). In this experiment using WT mice, individual differences among mice were large, and we therefore could not clearly confirm that the enhancement of brain inflammation was due to *P. gingivalis* infection, but we could confirm this tendency. Therefore, we believe that brain inflammation may be involved in the mechanisms by which cognitive function declined in the *P. gingivalis*-administered WT mice. These results also suggest that infection with *P. gingivalis* in WT mice reduces cognitive function but does not elicit AD-specific pathologies, such as Aβ increase. These findings suggest that *P. gingivalis* infection may be related to exacerbation of AD pathology rather than affecting the onset of AD. In an in vitro BBB permeability test, it was revealed that LPS may penetrate the BBB (Supplementary Fig. 4). The results suggest that J20 mice are more susceptible than WT mice to *P. gingivalis* and that inflammation is strongly induced and reflected in the blood. Inflammatory mediators in the blood enhance BBB permeability and may penetrate into the brain. Actually, the increases in inflammatory cytokine levels in the brain in APP-Tg mice were greater than those in wild-type mice after peripheral LPS injection.^34^ In addition, the permeability of the BBB was increased in APP-Tg mice by the peripherally evoked inflammation. These inflammatory mediators may activate microglia and promote Aβ production in cells.^35^ In addition, the activation of innate immunity with these factors in brain could affect AD pathology.^36^ Therefore, the increased innate immune response in the brain originating from the intraoral infection of *P. ginigvalis* may have exacerbated AD pathology. In the future, we will examine the detailed mechanism.

Periodontitis is a major cause of loss of teeth. Since there is a significant correlation between AD and tooth loss, it has been suggested that periodontitis may be a risk factor for AD.^37,38^ Tooth loss reduces chewing function, thereby reducing cerebral blood flow, and then cognitive function may deteriorate. Oue et al. reported that cognitive decline was observed in the APP transgenic mouse from which teeth had been removed, but deposition of Aβ and induction of brain inflammation were not observed.^39^ On the other hand, in this study, we found that APP transgenic mice that had been infected with *P. gingivalis* and had developed periodontal disease showed increased cerebral Aβ deposition and enhanced intracerebral inflammatory response, as well as a decrease in cognitive function. These results suggest that cognitive decline due to periodontal disease and that due to tooth loss are caused by different molecular mechanisms. Namely, tooth loss causes memory impairment in an amyloid-independent manner and is associated with a decreased number of neural cells in the hippocampus by reduction of BDNF signaling.^37^ We also found that chewing dysfunction caused by liquid diet induces memory impairment and hippocampal neuronal loss due to impaired BDNF cascade.^40^

An intervention study to test the efficacy of periodontal treatment on AD features are needed to conclude that periodontitis is truly a risk factor for AD; however, this study suggests a new possibility that periodontitis directly deteriorate the pathological features of AD. AD is a serious problem in the world; however, there is still no effective method for the prevention of AD or a fundamental method for treatment.^41^ Aβ deposition in the brain is initiated during the first half of the 40s, more than 15–20 years prior to the onset of cognitive impairment in AD patients.^42,43^ It is the period in which patients with periodontal diseases also begin to increase drastically. Because, periodontal infections are treatable, treatment of periodontal diseases during this period may be effective for delaying the onset or progression of AD.

## Methods

### Mice

Female hemizygous transgenic (hAPP-J20) and non-transgenic mice (WT) were obtained from the J20 line, which expresses hAPP containing both the Swedish and Indiana mutations under the control of a PDGF-β chain promoter.^44^ Mice were housed in a pathogen-free facility at a maximum of five mice per cage until the study began, at which time the mice were housed individually. The mice were kept on a 12-h light/dark cycle (lights on at 7:00 am) and, food and water were available ad libitum.^45^ All mice were bred and maintained in specific pathogen-free conditions in the animal facility at Aichigakuin University, and all experiments were performed in accordance with institutional guidelines of the Animal Experiment Committee and Gene Recombination Experiment Committee.

### Bacteria and culture conditions

Culture of *P. gingivalis* was performed according to previous reports.^31,46^ Briefly, *P. gingivalis* ATCC 33,277 was cultivated with 5% laked rabbit blood-supplemented brucella HK agar (Kyokuto Pharmaceutical Industrial Co., Ltd., Tokyo, Japan) with hemin (2.5 µg/ml), menadione (5 µg/ml), and dithiothreitol (0.1 mg/ml) and in Trypticase soy broth (BD, Franklin Lakes, NJ) with yeast extract (2.5 mg/ml), hemin (2.5 µg/ml), menadione (5 µg/ml), and dithiothreitol (0.1 mg/ml). Bacterial cells were collected, washed, and suspended in phosphate-buffered saline (PBS). The concentration of bacteria was determined with a spectrophotometer at an optical density of 660 nm. Live *P. gingivalis* (10^9^ CFU) was resuspended in 100 µl of PBS with 2.5% carboxymethylcellulose.

### Development of periodontitis in mice

A mouse model of periodontitis was established as described previously with minor modification.^46^ Briefly, sulfamethoxazole (700 μg/ml) and trimethoprim (400 μg/ml) in water bottles were given to 62-week-old J20 female mice at any time for 10 days. Three days after the treatment, 10^9^ CFU of live *P. gingivalis* in 100 µl of PBS with 2.5% carboxymethylcellulose was applied to the gingival margin of each mouse under brief isoflurane anesthesia. After inoculation with *P. gingivalis*, the mice were fasted for 1 h. PBS containing only 2.5% carboxymethyl cellulose (100 μl) was administered to the control group. Five weeks after the oral challenge, a novel objection test was performed. The mice were then sacrificed, and the sera and brains were collected. Maxillary specimens were also collected for micro-CT and histopathological studies.

### Evaluation of mandibular bone loss

To evaluate morphological changes in the alveolar bone, the mandibular alveolar bone was scanned by micro CT (R_mCT AX; Rigaku Corporation, Tokyo, Japan) in mice 11 weeks after inoculation of *P. gingivalis*. The computed tomography was set according to slice thickness (50 μm), voltage (90 kV), and electrical current (150 µA). Three-dimensional images were made using TRI/3D Bone (Ratoc, Tokyo, Japan). The distance from the mesial buccal cement–enamel junction to the alveolar bone crest of the second molar was measured as a reference for bone height. The percentage of bone resorption was calculated as the distance from the mesial buccal cement–enamel junction to the alveolar bone crest divided by the length of the root × 100.^47^

### Evaluation of cognitive function

The novel objection test was performed on mice to test cognitive memory as described previously.^48^ Briefly, in the training session, we placed two objects in a box (30 cm × 30 cm × 35 cm high). The objects were a white lamp and a square bottle, the shapes and colors being different but sizes being almost the same. The mouse was then placed in the box and search behavior for the two objects was recorded for 10 min. We defined a mouse searching for an object as a mouse facing an object, touching an object, or performing an action to sniff an object. After 24 h of the training session, one of the objects used during the training was replaced with a new object, and the mice were returned to the box. The mice were allowed to freely search for 3 min. The time spent searching for each object was recorded, and the duration was evaluated as the degree of cognitive function.

### Evaluation of Aβ deposition in the brain

The mouse brains were soaked in 4% paraformaldehyde at 4 °C for 24 h. After fixation in 4% paraformaldehyde, the brains were then embedded in paraffin and sectioned at 5 µm. The mandibular bone was soaked in 4% paraformaldehyde at 4 °C for 48 h. The brain sections were immunostained using anti-Aβ antibody 82E1 and visualized by the avidin–biotin–peroxidase complex procedure (Vectastain Elite ABC kit, Vector Laboratories, Burlingame, Canada) using diaminobenzidine (ImmPact DAB, Vector Laboratories) as the chromogen. Then the sections were observed with a microscope (OLYMPUS, Tokyo, Japan). The number of immunopositive pixels in the hippocampus were counted, and the values were converted to the area as the amount of Aβ deposition.^39^

### Measurements of Aβ oligomer, TNF-α, and IL-1β in the brain

Human oligomerized Aβ was measured using an ELISA kit purchased from Wako Pure Chemical Industries, according to the manufacturer’s instructions. Briefly, the right hemispheres from the brains of transgenic APP/J20 mice treated with or without *P. gingivalis* were collected and homogenized. Quantification of total protein was determined using a Micro BCA protein assay kit and was measured on an absorbance microplate reader (Molecular Devices, Sunnyval, CA). The homogenates were centrifuged at 100,000 ×  *g* for 20 min at 4 °C. Levels of TNF-α and IL-1β in the supernatants were measured by ELISA (R&D systems Inc., Minneapolis, MN) according to the manufacturer’s instructions. Insoluble materials in the sediments were sonicated in PBS with 6 M guanidine hydrochloride and were centrifuged at 100,000 × *g* for 20 min at 4 °C. The supernatant was then diluted 1:10 before carrying out the sandwich ELISA, which measures insoluble Aβ oligomer levels, but not amyloid monomers, as detailed in the manufacturer’s protocol. The final Aβ oligomer values were determined following normalization of total protein levels.^49^

### Detection of endotoxin in the brain

The endotoxin in mouse serum and brain lysate was measured by a Limulus assay using an Endospecy ES-50M kit (Seikagaku Co., Tokyo, Japan).^50^

### Measurement of TNF-α and IL-1β in microglia cultures

A 6-3 microglia cell clone culture kit (Cosmo Bio Co. Ltd., Tokyo, Japan) was used in this study. The microglial cell line 6-3 (2 × 10^4^ cells/cm^2^) was seeded in 96-well culture plates and incubated for 24 h. The cells were then stimulated with 10 µM Aβ 42 for 6 h and further stimulated with *P. gingivalis*-derived LPS (0.01, 0.1, 1.0 µg/ml, LPS-PG from Invitrogen, San Diego, CA) for 24 h. Levels of TNF-α and IL-1β in the media were measured using an ELISA kit (R&D Systems) according to the manufacturer’s instructions.^51^

### Measurement of Aβ oligomer in murine neuron cultures

Primary neuronal cultures were prepared from the cortices of embryonic day 17 C57BL/6 mouse embryos.^52^ Briefly, cortices were dissected and the meninges were freed. The cortical fragments were incubated with 0.25% trypsin and 20 µg/ml DNase I in phosphate-buffered saline at 37 °C for 15 min, dissociated into single cells by pipetting, and then resuspended in serum-free Dulbecco’s modified Eagle’s medium/Ham’s F-12 (Wako Pure Chemical Industries, Osaka, Japan) supplemented with N2 Supplement with Transferrin (Wako Pure Chemical Industries, Osaka, Japan) supplemented with N2 Supplement with Transferrin (Wako Pure Chemical Industries, Osaka, Japan).^53^ Primary neuronal cells were plated in poly-ethylene-imine-coated 12-well plates at a density of 1 × 10^6^ cells per well. The neurons were stimulated with or without 0.1, 1, 10 µg/ml of *P. gingivalis*-derived LPS for 24 h. Then levels of Aβ40 and Aβ42 in the media were measured by an ELISA (Wako Pure Chemical Industries).

### Statistics

Statistical analyses were performed using an unpaired Student’s *t*-test. Multiple comparisons were performed by one-way analysis of variance and the Bonferroni or Dunn method, with results presented as means ± standard deviation. *P*-values less than 0.05 were considered statistically significant.

### Approval of experimental protocol

All experimental protocols were approved by institutional guidelines of the Aichigakuin University and National Center for Geriatrics and Gerontology.

### Data availability

The data that support the findings of this study are available from the corresponding author upon reasonable request.

## Electronic supplementary material

supplementary materials

## Electronic supplementary material

**Supplementary information** accompanies the paper on the *npj Aging and Mechanisms of Disease* website (10.1038/s41514-017-0015-x).
