# Supplementary material for: Periodontitis induced by bacterial infection exacerbates features of Alzheimer’s disease in transgenic mice
Source: NPJ Aging Mech Dis. 2017 Nov 6;3:15. doi: 10.1038/s41514-017-0015-x (PMC5673943; doi:10.1038/s41514-017-0015-x)
Supplement: Supplementary file 1 — supplementary materials [file 41514_2017_15_MOESM1_ESM.docx]

**Supplementary Materials**

**Legends for Supplementary Figures**

Supplementary Figure 1. Evaluation of cognitive function in *P. gingivalis*-infected WT mice. Results are expressed as means ± S.D. (n = 6-7)

Supplementary Figure 2.

Aβ40 and Aβ42 in the cortex of *P. gingivalis-*infected WT mice. Concentrations of Aβ40 and Aβ42 in extracts of the cortex were measured by ELISA. Control: Mice inoculated with CMC alone, *P. gingivalis*: Mice inoculated with CMC and *P.gingivalis.* Results are expressed as means ± S.D. (n = 6-7)

Supplementary Figure 3. TNF-α and endotoxin in serum in *P. gingivalis-*infected WT mice. Amounts of TNF-α in serum were measured by ELISA. Levels of endotoxin in serum were measured by the Limulus assay. Results are expressed as means ± S.D. (n = 6-7)

Supplementary Figure 4. Permeability of LPS in an *in vitro* BBB model. *P. gingivalis* LPS (1.0 and 10 µg/ml) or sodium fluorescein (Na-F) was applied into the inserts of a BBB kit ( PharmaCo-Cell Co. Ltd., Nagasaki, Japan) and incubated for 24 h. Then, the amounts of Na-F and LPS that had penetrated into the lower chambers were determined. The permeability coefficients (Papp) were determined according to the instruction of the manufacturer. Results are expressed as means ± S.D. (n = 3)

Supplementary Figure 1

Supplementary Figure 2

Supplementary Figure 3

Supplementary Figure 4
